# Medical News and Miscellany

**Published:** 1887-10

**Authors:** 


### MEDICAL NEWS AND MISCELLANY.

DR. W. W. WALKER'S son is attending lectures at the Tulane, and Dr. J. W. McLaughlin's son is at the College of Physicians and Surgeons, New York.

DR. C. M. HARRISON, of Del Rio, has been appointed surgeon of the S. P. Railway at El Paso, and has removed to that point.

DENGUE.—The dengue has recently prevailed as an epidemic in Albany, Shackleford county, Texas, sparing neither age, sex nor previous condition. Our friend, Dr. Powell, is a convalescent.

DR. J. M. BALL, of New Boston, Texas, has a son attending the University of Texas, and Dr. A. L. Patton, of Minneola, has a daughter there, also.

DR. D. V. SPRING, of Lyons, Texas, is just convalescent from a long spell of sickness, which has confined him to his room over five months, and the first day he sat up he was attacked with erysipelas! The doctor has our sympathy, and best wishes for his recovery.

FOR BURNS.—Dr. W. C. Wile finds the colorless *Ext. Pinus Canadesin*, (Rio Chemical Company), a most admirable dressing for burns and scalds, possessing soothing and healing properties in an eminent degree.

WE understand that the St. Louis Post Graduate Medical School is literally booming this fall. It ought to; and we know of at least a dozen Texas physicians who are going to the New York Post Graduate soon.

A LESSON, LEARNED IN TEXAS (?)—Most members of the T. S. M. A. will remember Dr. Wyeth's not very complimentary remarks at Tyler, when Dr. Will Davis' paper on the treatment of hæmorrhoids by carbolic injection was read. *Now* Wyeth is quoted by Prof. Gerster as authority for the treatment!

---

YELLOW FEVER IN FLORIDA.—The disease has entirely disappeared from Key West, but has made its appearance at Tampa, and one case has been reported at Palatka. The season is so far advanced that there is scarcely a possibility of the fever becoming epidemic, or spreading beyond its present limits; however, it is one of those things which it will not do to Tampa with.

---

THE OXFORD RETREAT, a Sanitarium in, or near Cincinnati, has lately gained an unenviable notoriety in consequence of certain newspaper publications, in which it is alleged that a Dr. Cook in charge, had, for pay, cooperated with relatives of a rich young man from Memphis, to imprison him, in order that said relatives might get posssesion of his property. The young man it seems, had voluntarily placed himself under the treatment of Cook, as he says: "to get the whisky out of him." When it was all out, *he* tried to get out, but Cook said "No sir, you stay here." All his letters were subject to the eye of Cook, but he smuggled one out, and enlisted the aid of friends who procured his release; and now comes the tug of war,—an indictment perhaps, of the above parties. We await developments.

---

### THE STATUS OF THE LUNATIC ASYLUM TROUBLE.

It will be remembered that the Superintendent refused to step down and out, on invitation of a majority of the Board of Directors, on the finding, as reported, of guilty, on certain charges, and that he was sustained, on legal opinion that it requires a full board to depose him, even for cause. In this opinion, we learn, the Governor concurred. Hereupon, Dr. Smoot, one of the majority members, resigned. A Mr. Hirshfeld was appointed in his stead. It will be remembered also, that the original Board had promised employees who had testified against Dr. Dorsett, that they should not lose their places in consequence of that act. At a late meeting of the Board, some six employees were discharged, (on various charges by the Superintendent, some "lazy," some "incompetent," etc.) and it is a mere *coincidence* that all of them had testified aginst the Superintendent at the late trial. Two of the members of the Board, Mr. Castleman and Mr. Deffenbaugh, opposed their discharge, and reminded the Board of the promises given these people, but the new member, Mr. Hirschfeld, voted with what had been, previously, the minority, Dr. Howard and Mr. Archer, and out the employees went. Thereupon, Messrs. Castleman and Deffenbaugh resigned. Thus, in this vexatious matter the *minority* has not only "ruled," but seems to have ousted the majority; they are *out*, anyhow,—and the Superintendent is "in."

It seems that sauce for the goose is *not* sauce for the gander, any longer. This is an age of progress. For hundreds of years that maxim had held undisputed sway, and was applied in all ordinary affairs; but now it is different, and circumstances alter cases." That is to say, the Board ruled, and was sustained, that a full vote is necessary to remove a Superintendent, but a bare majority can remove any other officer. Consistency is said to be a jewel;—it's more; it's a "daisy," a "gem of purest ray serene;" it's "scarce."

After all, this seems to be in accordance with a kind of streak in human nature, that prompts one to an instinctive discrimination as to *rank*. We remember during the war, a lieutenant was entitled to one cord of wood, a Captain two, a Major three, and a General five. Realizing that it takes no more wood to warm a General, than it does to warm a Captain, (*cœteris paribus*), we never could understand this discrimination: but we have seen the same rule applied in so many other relations of life, that one may come to formulate a sort of rule of action; thus, a nod to an acquaintance, a bow to a banker, ditto, and tile-tilt to a Senator, or a cattle king, and a regular *salaam* to a Govervor, or any other high-upper;—thus, also, a miserable little worm for a sucker, or cat,—a fly for a perch, but a two-dollar and a half Buel-spinner is set out in deference to the festive trout, the upper-ten-ner of the piscatoral tribe, and lord of the watery world.

---

Dr. E. W. Walton, of Texas, resident physician and first assistant at the clinic of Manhattan Eye and Ear Hospital, New York, is on a brief visit to his friends, and father's family in Austin. The doctor is as handsome as ever, and still a bachelor.

THE EDITION DOUBLED.

I certify that my contract with Dr. Daniel is for 2000 copies of the JOURNAL monthly, and that 2000 copies of this, the October, '87 edition has been printed by this establishment.

EUGENE VON BOECKMANN,
Steam Printer, Austin, Texas.

Referring to the above, we claim to have the best medium for reaching and impressing the physician of Texas, extant. Our advertisers are all but unanimous in acknowledging the value of the JOURNAL; and as evidence of their appreciation, and of its success in answering their ends, we cite with pride, the fact, that nearly all of our patrons have renewed their contracts at advanced rates,—from 25 to 100 per cent. We return our grateful acknowledgments to both, advertisers and subscribers, for the very generous support extended the JOURNAL, whereby we have been enabled to increase the size 20 pages, and to double our monthly edition.

---

THE LATEST RIVAL TO COCAINE.

A number of articles recording the results obtained by experimenters, who have investigated the anæsthetic virtues of gleditschine, the new local anæsthetic derived from *gleditschia tri acanthos*, and which was at first improperly called stenocarpine, have appeared in current medical literature.

It seems probable that we have in this alkaloid derived from a plant indigenous to this country, a valuable addition to our local anæsthetics. Whether it will supplant cocaine remains to be proven.

We learn that Parke, Davis & Co., are thoroughly investigating it at their laboratory. They announce that they are prepared to supply samples of the fluid extract of *gleditschia tri acanthos* for clinical trial, together with a working bulletin containing the reports that have been published to date.

---

THE LATE DR. JOHN H. MORTON, OF DALLAS.

Under the head of "Noble Tennesseian," the *Nashville Medical News* pays a handsome tribute to the late distinguished surgeon, Dr. John H. Morton, and relates a fact in his career, which we believe was not generally known, to-wit :

"He then [after graduating at the University of Pennsylvania, 1853], took a course of lectures at the College of Surgeons, Paris. Having in a few months acquired the diplomas of that famous institution, he accepted, early in the Crimean war, a commission as assistant surgeon in the Russian army, in which he rendered such distinguished service at the hospitals in the field, that at the close of the war, he was knighted by the emperor, and received at the hands of that potentate, the decoration of the order of St. Ann."

---

DR. A. G. PENDLETON, of San Marcos, Texas, has just returned from an European tour, during which he visited Ireland, Scotland, England, France, Belgium, and a part of Germany. A large part of his time was spent in the hospitals, especially the Royal Infirmary of Edinburgh, the St. Thomas and King's Hospitals, London, Hotel Dieu at Paris, etc. During his stay in London, Dr. Pendleton was made a "F. R. C. S.," a distinguished compliment to an American physician, and especially a Southerner.

---

A FORGERY.—The name of the late Dr. W. J. Burt, Secretary Texas State Medical Association is paraded, attached to a very illiterate "certificate" or "testimonial" to the virtues of a certain so-called "Panacea," now being peddled by a "Doctor" Goodman, in Texas. We have the authority of Dr. Burt's sons for saying that it is a forgery, and that the name of their father is used in that connection without authority.

---

"The *Medical Student* says: Insane Asylum abuses are being discussed by a majority of Medical Journals from the *New York Medical Record*, to *Daniel's Texas Medical Journal*." We wonder if DANIELS appreciates the tail-end position."

*St. Louis Weekly Review.*

It is very evident that "*Medical Student*" meant that the subject was being discussed by leading Medical Journals, from New York to Texas, those States representing the extremes of the country, and being represented by the *Record*, and *Daniel's Texas Medical Journal;* though his idea was awkwardly expressed. As we are in blissful ignorance of who or what "*Medical Student*" is, whether a publication by that name, or a contributor under that *non de plume*, did we believe he, she or it could be so stupid as to assign, a "tail-end" position to a *leading* Medical Journal, we would pity him, her or it; or, if it were intended as a slur, we would reply to the above query in the language of the gentleman who was *kicked at* by a jas-sack,—"consider the source." Next.

THUS FAR AND NO FARTHER.

State Health Officer Rutherford, with his accustomed vigilence, pounced upon a cargo of lemons and oranges at the gates of Texas, which had passed the quarantine of New York, and had there been re-shipped on a Mallory Line steamer for Galveston.

The vessel that brought this cargo from Italy to New York, had, in all probability, like the "old ship of Zion," "landed many thousands, and will land as many more," of these luxuries, and they have been distributed to every village, town and city on this great American continent. The health officers, everywhere, save in our own great State, have seemingly displayed a marvelous indifference to the dangers thereby incurred,—the danger of turning loose myriads of Italian or Sicilian microbes of all sorts, sizes and descriptions, on American soil.

When we reflect that lemonade is one of the favorite beverages of the *comma bacillus* (according to Koch; see reports of successful "cultures" on a slice of lemon pie,—in the *Berliner Wochenschrift* and the *Centralblatt Kinderheilkunde*), and that all forms of micro-organisms,—from the *Cryptococcus Xanthogenicus* of Friere, to the giant *coccus anthropophagous*, or man-eater, have a predilection for tropical fruits, amounting almost to a mania, and indeed, Koch, (who is an enthusiast), is quoted as saying in his emphatic way, that if there is anything on this green earth that a microbe does dote on, it is a Messina orange),—we must infer that these cargos are dangerous, and we feel that too much censure can not be heaped upon those criminally negligent guardians of the public health. The people of Texas meanwhile are to be congratulated on their splendid isolation from these dangers; nor need their dreams of security be disturbed by visions of the dreaded coccus nestling 'mid the golden groups of the luscious "banans,"—nor clinging to the cortex of the toothsome orange;—for like King Canute to the sea, the fiat goes forth, "thus far shalt thou come, and no farther."

